# Supplementary material for: Metabolome and Transcriptome Joint Analysis Reveals That Different Sucrose Levels Regulate the Production of Flavonoids and Stilbenes in Grape Callus Culture
Source: Int J Mol Sci. 2024 Sep 27;25(19):10398. doi: 10.3390/ijms251910398 (PMC11476901; doi:10.3390/ijms251910398)
Supplement: Supplementary file 1 [file ijms-25-10398-s001.zip › ijms-3191186-figures.pdf]

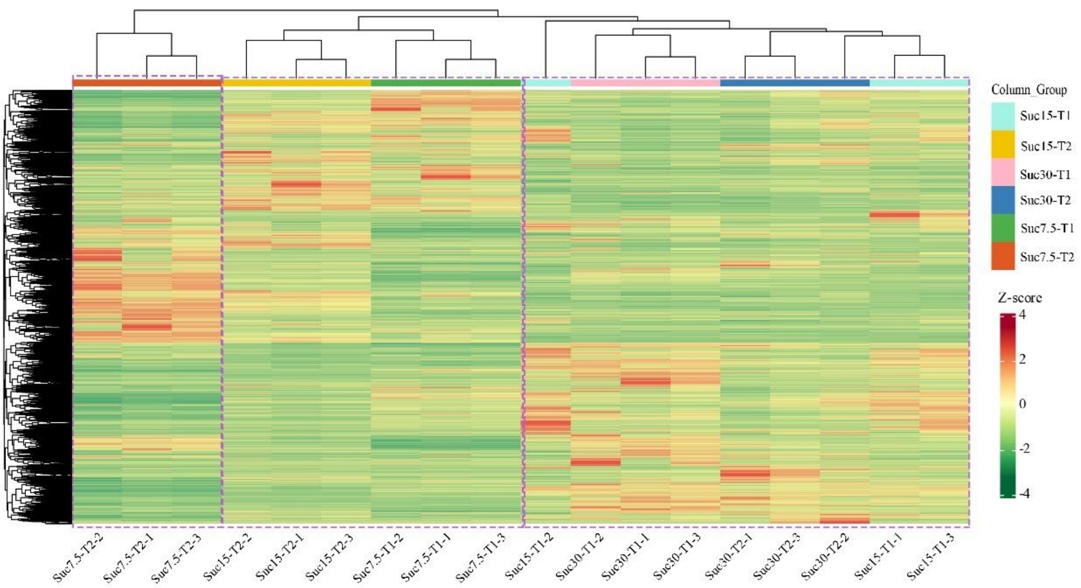

Figure S1. Differential metabolites hierarchical classification.

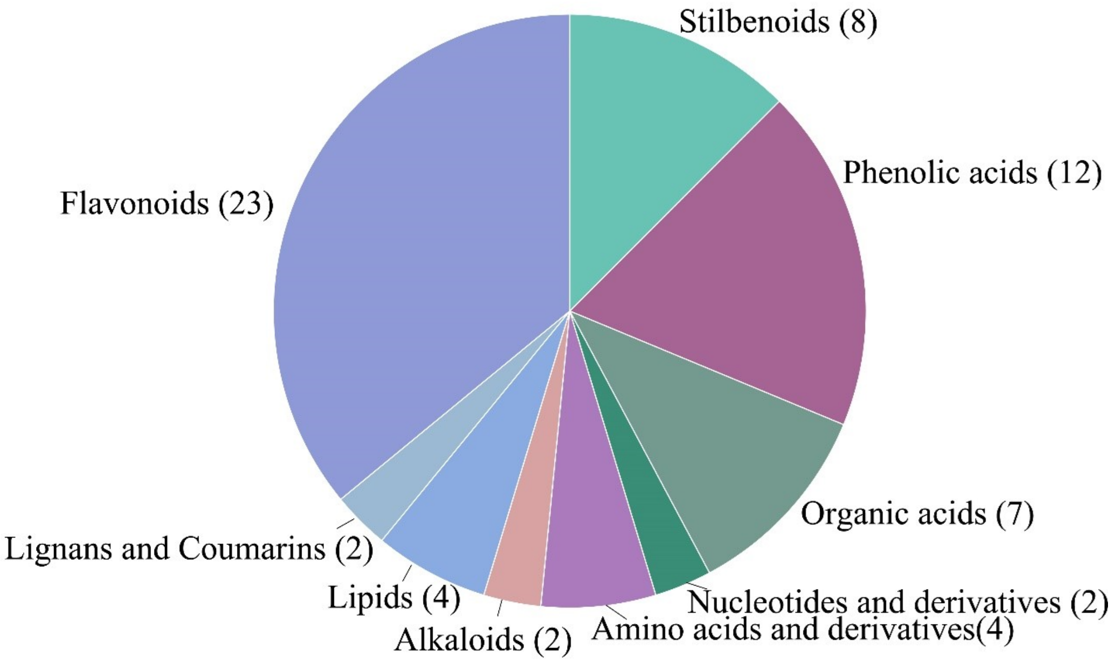

Figure S2. The pie chart of differential metabolites in the "T1 vs T2" comparison group.

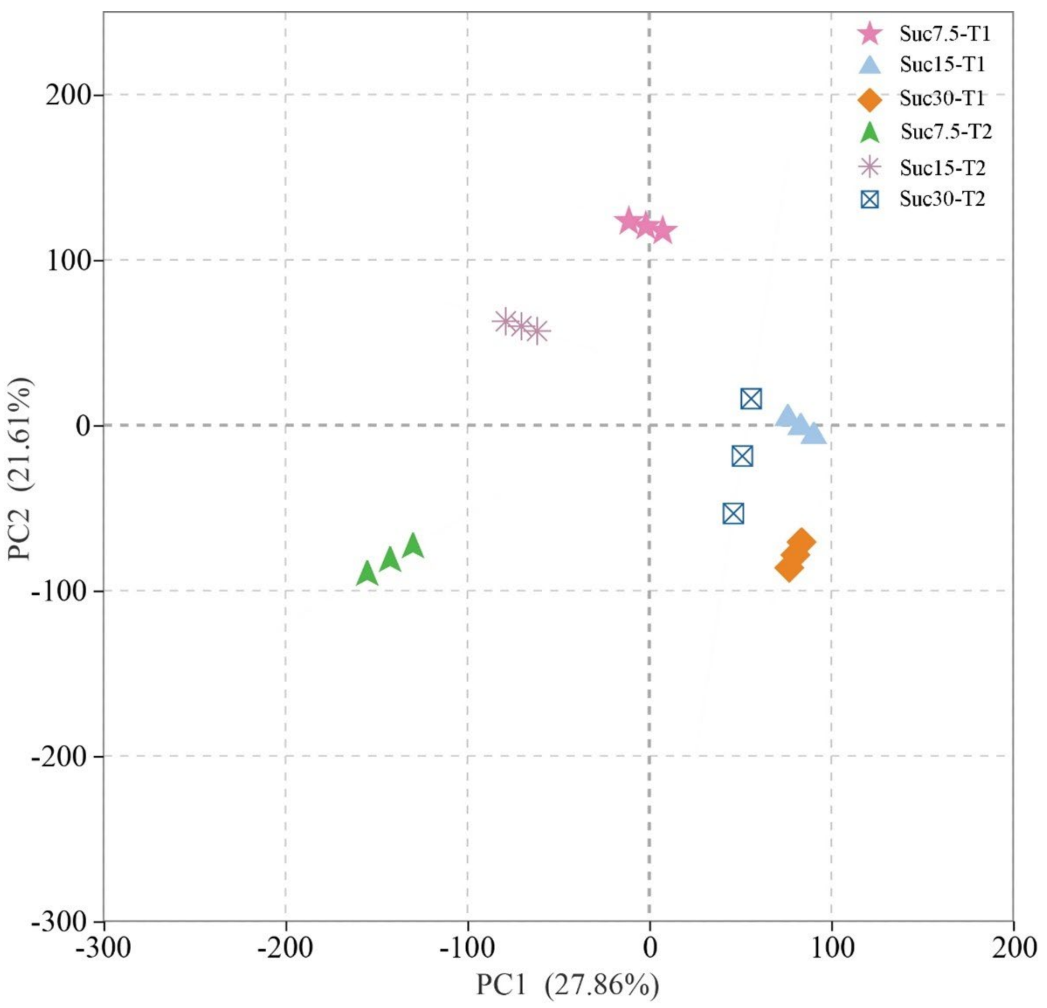

Figure S3. PCA analysis for the transcriptomic data among the grape callus samples.

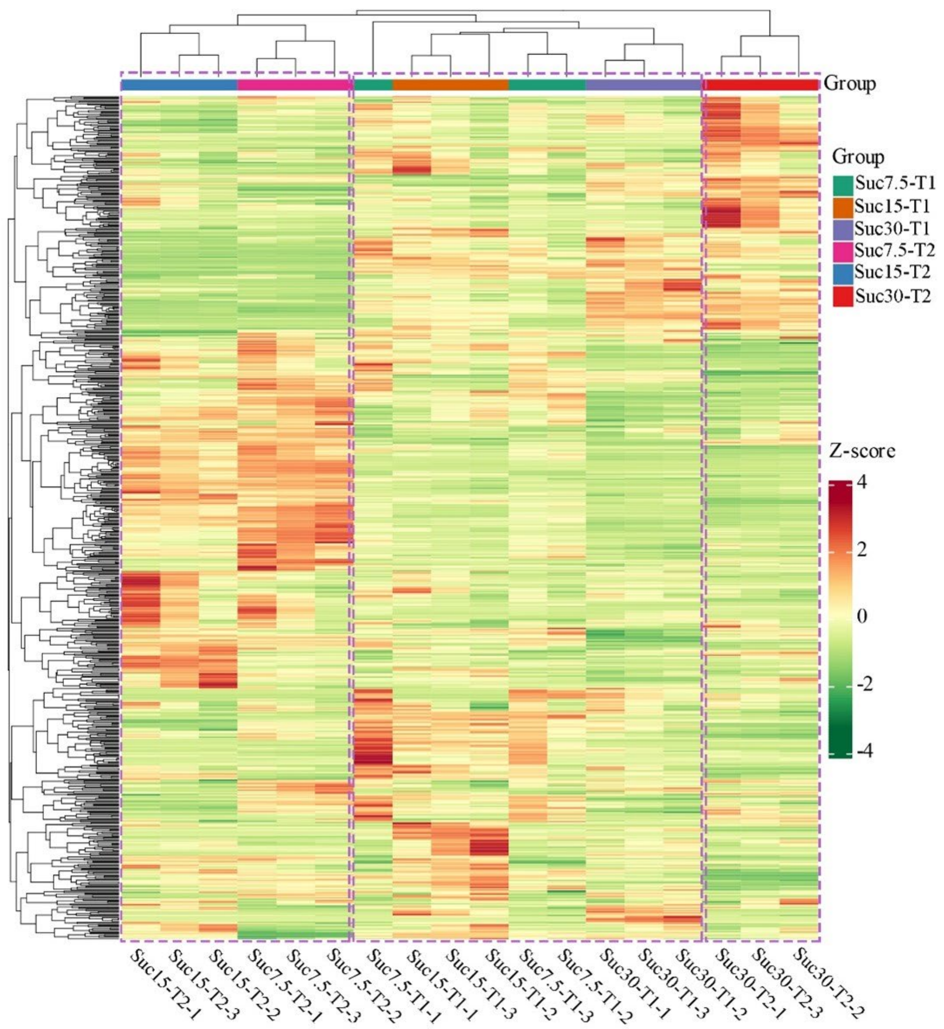

Figure S4. Cluster analysis of the transcriptomic data among the grape callus samples. The color indicated the relative levels of genes from low (green) to high (red).

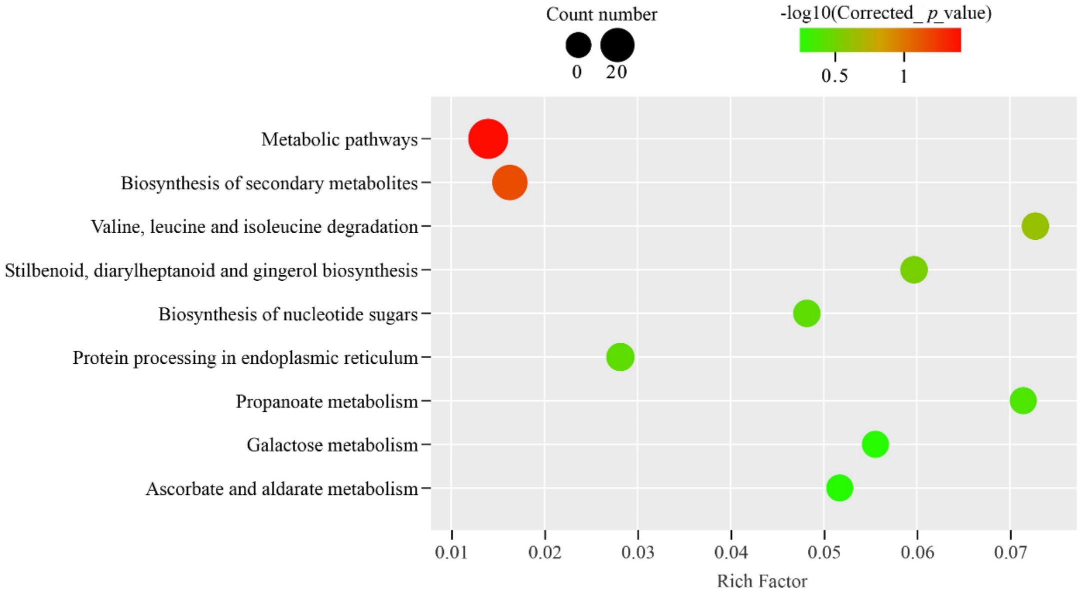

Figure S5. KEGG enrichment analysis of DEGs in the comparison group "T2".

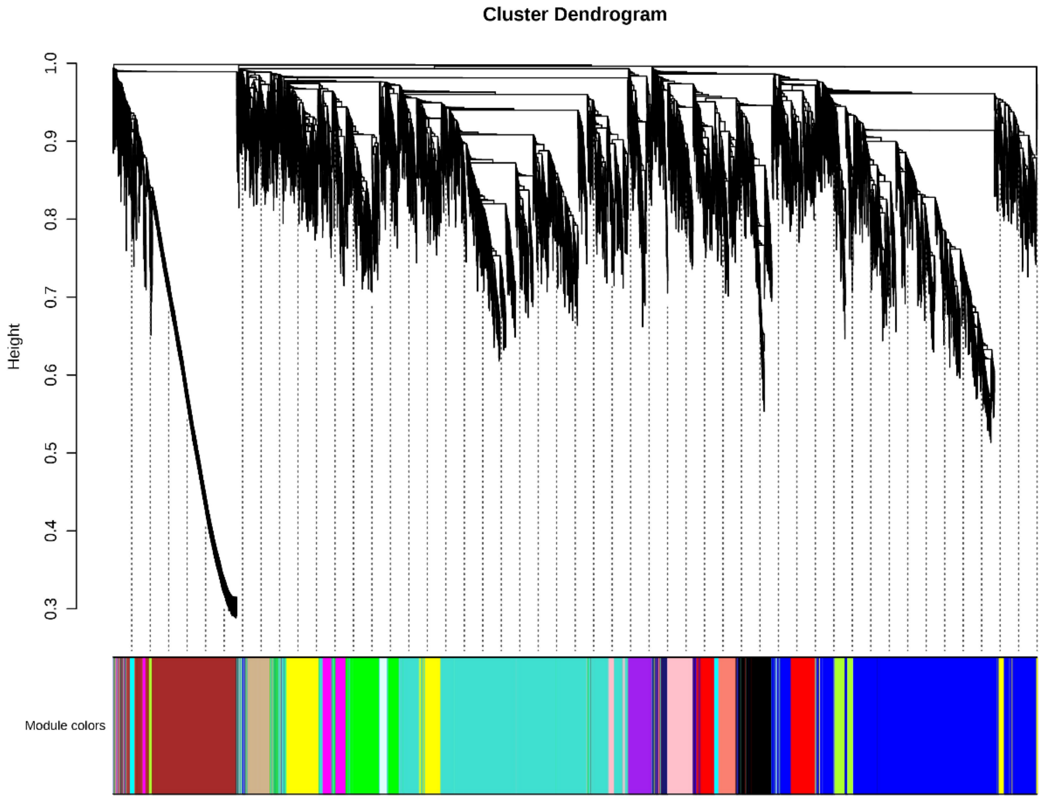

Figure S6. Co-expression modules analysis using WGCNA. The same color represents the same module.
